# A pilot feasibility study of gabapentin for managing pain in children with dystonic cerebral palsy

**DOI:** 10.1186/s12887-021-02847-1

**Published:** 2021-08-28

**Authors:** Adrienne Harvey, Mary-Clare Waugh, James Rice, Giuliana Antolovich, Lisa Copeland, Francesca Orsini, Adam Scheinberg, Clare McKinnon, Megan Thorley, Felicity Baker, George Chalkiadis, Kirsty Stewart

**Affiliations:** 1 Murdoch Children’s Research Institute, 50 Flemington Road, 3052 Parkville, Australia; 2 Royal Children’s Hospital, Melbourne, 50 Flemington Road, 3052 Parkville, Australia; 3 Neurodisability and Rehabilitation, Murdoch Children’s Research Institute, 50 Flemington Road, VIC 3052 Parkville, Australia; 4 The Children’s Hospital at Westmead, Cnr Hawkesbury Rd &, Hainsworth St, 2145 Westmead, Australia; 5 Women’s and Children’s Hospital, 72 King William Rd, 5006 North Adelaide, Australia; 6 Queensland Children’s Hospital, 501 Stanley St, 4101 South Brisbane, Australia

**Keywords:** Dystonia, Cerebral palsy, Gabapentin

## Abstract

**Background:**

Gabapentin is often used to manage pain in children with dystonic cerebral palsy, however the evidence for its effectiveness in this population is limited. The primary objective of this feasibility pilot study was to assess the factors which might impact on a future randomised controlled trial including the ability to recruit and retain participants, assess adherence/compliance to the prescribed intervention, and ability to complete all outcome assessments. The secondary objective was to gather preliminary evidence for the effectiveness of gabapentin at reducing pain, improving comfort and reducing dystonia in children with dystonic cerebral palsy.

**Methods:**

This open label pilot study recruited children aged 5–18 years with dystonic cerebral palsy and accompanying pain affecting daily activities from four centres around Australia. Children were prescribed gabapentin for 12 weeks and were assessed at baseline, 6 weeks and 12 weeks. The primary outcome was feasibility of the protocol. Secondary outcomes were pain behaviour, pain intensity, care and comfort, individualised goal setting and dystonia severity.

**Results:**

Thirteen children (mean age 10.4 years (SD 2.4yrs), 9 females) were recruited from 71 screened over 15 months. Two children withdrew while eight children experienced side effects. There were issues with adherence to medication dosage regimens and data collection. Improvements were seen in pain behaviour, comfort and pain related goals at 12 weeks. Dystonia was not significantly changed.

**Conclusions:**

Whilst gabapentin has potential to improve pain and comfort in children with dystonic CP, the feasibility of implementing a definitive randomised controlled trial is low. Alternative trials designs are required to further examine the effectiveness of gabapentin in this heterogeneous population.

**Trial registration:**

The trial was registered with the Australian Clinical Trial Registry (ACTRN12616000366459) on 22/03/2016 and the Therapeutic Goods Administration (CT-2016-CTN-00500-1) on 22/06/2016.

## Background

Pain is common in children with cerebral palsy (CP), with chronic pain the most commonly reported physical comorbidity of CP throughout the lifespan [1]. Pain prevalence in this population varies between 14 and 76 % due to inconsistent measurement, varying recall periods, and different participant age ranges between studies [2]. In children with CP, pain increases with increasing severity of gross motor impairment and age and is more prevalent in females [2–5]. Under-recognition of pain is common in children with CP [6] and likely results in inadequate pain management for many with significant impact on participation, social-emotional wellbeing and quality of life [2].

Dystonia, a movement disorder characterised by involuntary movements [7], has been cited as one of the most frequent causes of pain in children with CP [4]. The complex interplay between dystonia and pain renders it difficult to unravel the cause and effect for children with dystonic CP who have significant pain. Oral medications are frequently used as first line medical management for targeting dystonia in CP [8]; however, the evidence for their effectiveness is limited and side effects are common [9]. For children with dystonic CP who experience chronic pain, targeting the pain might not only improve pain, but also reduce the frequency and severity of dystonia.

Gabapentin is used frequently in the management of children with dystonic CP [8], however, there is little evidence to support its use for managing pain specifically in this population [9]. A retrospective observational study of gabapentin for severe dystonia in 69 children, 25 of whom had CP but whose results were not reported separately, found a significant decrease in the severity of dystonia and significant improvements in sleep quality, sleep amount, mood, pain, general muscle tone, involuntary muscle contractions, and seating tolerance [10]. More evidence is required specifically for managing pain in children with dystonia in CP.

This lack of evidence for the efficacy of gabapentin in pain management in children with dystonic CP strongly justifies the need for further prospective studies. Before designing a randomised controlled trial for this purpose, a pilot study is necessary to assess the feasibility of running a larger trial. The primary objective of this feasibility pilot study was to assess the factors which might impact on a future randomised controlled trial including the ability to recruit and retain participants, assess adherence/compliance to the prescribed intervention, and ability to complete all outcome assessments. The secondary objective was to gather preliminary evidence for the effectiveness of gabapentin at reducing pain, improving comfort and reducing dystonia in children with dystonic CP.

## Methods

This open label pilot feasibility study aimed to recruit children with dystonic CP who received gabapentin as treatment for pain. Children were screened for eligibility and assessed at baseline prior to commencing gabapentin for 12 weeks. Follow-up assessments occurred at 6 and 12 weeks.

 The study received ethics approval and governance authorisation through the Royal Children’s Hospital Human Research Ethics Committee (Number 36037D) and governance at the three other recruiting sites. Informed consent was obtained for all study participants via the parent, legal guardian, or person with power of attorney. The trial was registered with the Australian Clinical Trial Registry (ACTRN12616000366459p) on 22/03/2016 and the Therapeutic Goods Administration (CT-2016-CTN-00500-1) on 22/06/2016.

### Participants and recruitment

Children were recruited from the Rehabilitation and Developmental Medicine clinics of four Australian tertiary care centres between November 2016 and October 2018. Children aged 5 to 18 years, diagnosed with CP of all Gross Motor Classification Function System (GMFCS) levels with severe generalised dystonia (with or without spasticity) and chronic pain affecting daily activities were eligible to participate. Potentially eligible children were screened using: the Hypertonia Assessment Tool (HAT) [11] to confirm presence of dystonia, the Barry Albright Dystonia scale (BADS) [12] to measure severity of dystonia, and the Health Utilities Index 3 Multi-Attribute Health Status Classification System [13] (HUI 3) to quantify baseline pain. Children with a BADS score of 15 or higher in total, or 4 in one limb, and who scored III, IV or V for pain on the HUI 3 were eligible. Children were required to have no changes to medications that could influence dystonia in the previous three months.

Children were excluded if they: were currently receiving gabapentin or had been taking this in the previous three months, had orthopaedic surgery in the previous six months, demonstrated hypersensitivity to gabapentin in the past, or were currently taking other medications that interact with gabapentin (i.e. antacid, cimetidine, and opioids).

### Outcome measures

Descriptive characteristics of the participants included age, weight and height, predominant motor type and functional classification using the GMFCS, Manual Ability Classification System (MACS), and Communication Function Classification System (CFCS).

Primary outcome. Study feasibility was measured by recruitment numbers, withdrawals, completed numbers, numbers of side effects or adverse reactions and adherence/compliance to treatment as prescribed. Parents completed daily medication logs recording dosage of medication given, any side effects or adverse events and other relevant information.

Secondary outcomes. Pain was measured directly using the Paediatric Pain Profile (PPP) [14] weekly and the Faces Pain Scale- Revised (FPS-R) [15] at baseline, 6 weeks and 12 weeks. Comfort, health status and wellbeing were measured at baseline, 6 weeks and 12 weeks using the Caregiver Priorities and Child Health Index of Life with Disabilities (CPCHILD™) questionnaire [16] and the Care and Comfort Hypertonicity Questionnaire (CCHQ) [17]. Pain related goal setting with the child and family was conducted at baseline and 12 weeks using the Canadian Occupational Performance Measure (COPM) [18]. Dystonia severity at 6 weeks and follow-up was measured using the BADS.

The PPP is a standardised caregiver-report measure which scores child behaviours related to pain in children, including those with neurological impairments and communication difficulties. The FPS-R is designed for children aged 3 years and older who are able to self-report [15].

The CPCHILD™ is a reliable and valid measure of caregivers’ perspectives on the health status, comfort, well-being, and ease of caregiving of children with severe developmental disabilities [16]. The CCHQ is a caregiver questionnaire which rates the degree of difficulty experienced across personal care, positioning/transferring, comfort and interaction/communication [17].

The COPM is an individualized, client-centred outcome measure of a change in a client’s self-perception of occupational performance over time. It uses semi-structured interviews to measure child or parent perceptions of the child’s ability to perform tasks within their daily lives and their associated level of satisfaction with the performance of those tasks [18].

The BADS measures the severity of dystonia in CP in eight body regions [12]. Children were videotaped as they performed a number of actions and functional activities and the BADS score determined from these videos at a later stage.

### Procedures

Screening, descriptive information and assessments were all performed by an experienced physiotherapist or occupational therapist familiar with the tools and trained to ensure consistency of measurement. The HUI 3, COPM and the ‘Pain on a good day’ section of the PPP were collected at baseline and 12 weeks. All other outcome measures were collected at baseline, 6 weeks and 12 weeks.

Due to a lack of available dosing guidelines for gabapentin in children with CP, a standardised dosing regimen was developed for this study based on a previous clinical audit of doctors [8]. It consisted of a starting dose of 100 mg daily increasing gradually over the first 5 weeks to reach 300 mg three times daily in week 5. The dosage was increased over an additional 1–2 weeks for those children weighing greater than 30 kg and where the dosage of 30 mg/kg/day was not enough to effect change. (week 6–350–450 mg three times daily and week 7–400–500 mg three times daily). In addition, the dosage was escalated beyond 300 mg three times daily if there was no change in symptoms and the child tolerated the medication at that dose and a dose of 50 mg/kg/day had not been exceeded.

### Data analysis

Screening and baseline characteristics of participants were presented using means and standard deviations (SD) for continuous data (or medians and inter-quartile ranges for non-normal data) and proportions for categorical data. The primary outcome of feasibility included all participants screened and enrolled in the study. Adherence to medication dosing was determined from the medication log of each participant. The number of days (and the percentage of time over the 84-day treatment period) in which doses were taken according to protocol, following adjustment by clinician, or with dosage not specified were calculated. The numbers of days (and % of treatment period) in which the medication log had completely, or partially missing entries was also calculated. Means and SDs were calculated for each of these outcomes.

The secondary outcomes of efficacy of gabapentin at 6 and 12 weeks were presented using means and SD for continuous data (or medians and inter-quartile ranges for non-normal data) and proportions for categorical data. Individual change scores from baseline on the COPM, CP CHILD, CCHQ, PPP and BADS at 12 weeks were calculated and reported as means with their 95 % confidence intervals. The range of change scores for each outcome measure was reported for the group. In particular for the COPM, the mean performance score and the mean satisfaction score of the three tasks selected by the parents were calculated for each participant, so that each participant had two values, one for satisfaction, and one for performance.

## Results

### Recruitment

Across the four centres, 71 children were screened for eligibility. Of these, 51 were already taking gabapentin and 5 children/families lived at a distance too great from the tertiary centre to be able to fully participate in the study. None of the children had orthopaedic surgery in the previous 6 months or were on medications that could interfere with gabapentin. As a result, 15 children were eligible and approached, of which 13 agreed to participate and were enrolled. The 13 participants, ranging from 6.1 to 14 years, were recruited over 15 months. One child withdrew and another child discontinued medication after the first week on parent request. Data were collected at baseline on all 13 recruited participants, and at 12 weeks on 11 participants. The demographics of the included children are shown in Table 1. Children were classified primarily within GMFCS levels IV (*N* = 6) and V (*N* = 5) with predominant dystonia (*N* = 6), or mixed dystonia/spasticity (*N* = 7).

**Table 1:** Demographic data for the included children

	Total *N* = 13
Sex	
Male	4 (31 %)
Female	9 (69 %)
Age (years)	10.4 (2.4)
Epilepsy	5 (38.5 %)
Poor nutrition	1 (7.7 %)
Respiratory issues	1 (7.7 %)
Distribution of dystonia	
Quadriplegia	13 (100.0 %)
Movement disorder type	
Dystonia	6 (46 %)
Mixed dystonia/spasticity	7 (54 %)
GMFCS	
Level I	0
Level II	0
Level III	2 (15 %)
Level IV	6 (46 %)
Level V	5 (39 %)
MACS	
Level I	0
Level II	1 (8 %)
Level III	2 (15 %)
Level IV	2 (15 %)
Level V	8 (62 %)
CFCS	
Level I	4 (31 %)
Level II	1 (8 %)
Level III	2 (15 %)
Level IV	3 (23 %)
Level V	3 (23 %)

 Medication administration data was limited by several parents not completing the medication logs fully (only one parent completed it fully) or not returning the diary (four participants). Adherence to the treatment/dosing schedule of completed and submitted logs is presented in Table 2. On average, children took their doses either as per protocol or as per clinician adjustment or it was not specified for 67 % of their treatment period (56.3 days on average). On average, children missed doses or doses were not reported for 33 % of their treatment period (27.7 days on average). Across participants, on average, dosing schedules were modified 14.3 % of the time by doctors to accommodate side effects seen at higher doses.

**Table 2:** Adherence to treatment for the 11 children who completed the study

	Days	% treatment period
	Mean (SD)	Mean (SD)
Total treatment period length in days	84	100%
Total days in which doses were taken (either according to protocol dosage or following adjustment by clinician or not specified)	56.3 (38.1)	67.0% (45.4%)
According to protocol	33.6 (32.4)	40.0% (38.6%)
Following adjustment by clinician	12.0 (15.7)	14.3% (18.7%)
Dosage not specified	10.6 (19.7)	12.7% (23.5%)
Total days in which doses were missed (or dose not reported)	27.7 (38.1)	33.0% (45.4%)

### Side effects and adverse events

Eight of the 13 participants experienced side effects with a total of 22 side effects and two adverse events reported. Four participants experienced one side effect each, one participant experienced four side effects, two experienced five side effects each, and one experienced six side effects. The most common side effects were gastrointestinal issues, drowsiness/fatigue, weakness, and behavioural changes/irritability. Seven of the side effects reported were considered moderate and 15 mild. None of the side effects required treatment, with 17 resolving by the end of the study. One child withdrew from the study due to side effects, including lethargy, global weakness, and low mood. Two children reported adverse events which were considered severe; one child suffered a focal seizure which was considered not related to the medication and one child had behavioural and mood changes which was considered possibly related to the medication.

### Measures of efficacy

**Fig. 1:**
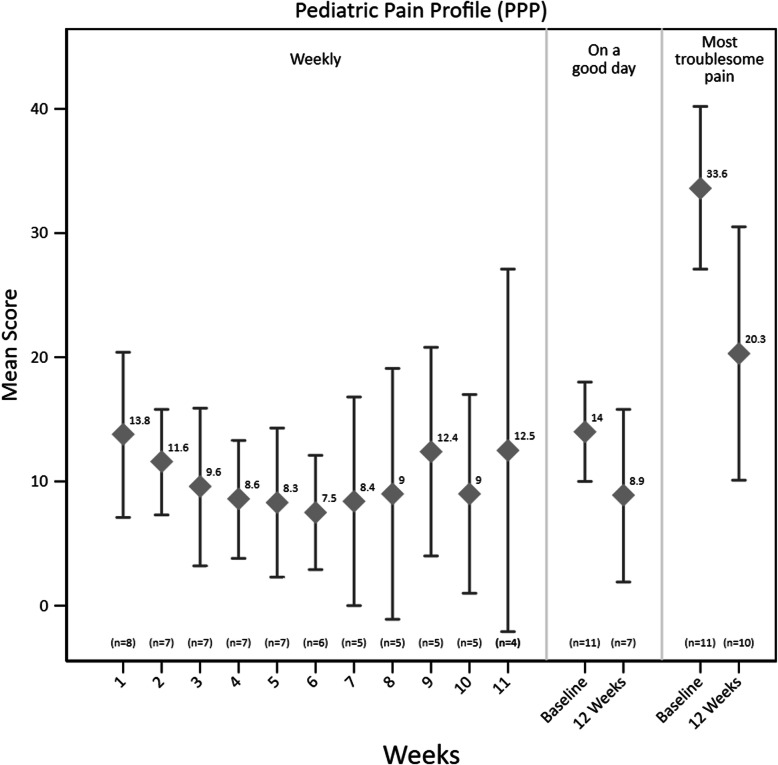
Weekly mean scores (SD) for Paediatric Pain Profile for all children over the 12 weeks and means scores (SD) for baseline compared to 12 weeks for “Pain on a good day” and “Most troublesome pain”

Adherence to the data collection and results for the PPP are shown in Fig. 1 while Table 3 shows results and adherence for all other secondary outcomes. There was good adherence to the PPP at baseline with 11 completed for most troublesome pain and pain on a good day, and at 12 weeks with 10 completed for most troublesome pain and 7 for pain on a good day. However, a number of weekly PPP questionnaires were incomplete with a range of 4–8 completed each week amongst the 11 children. For the remaining secondary outcomes there was good adherence at baseline and 12 weeks, but poorer adherence at the 6-week time point. At 12 weeks the most clinically significant findings were improvements in COPM performance and satisfaction scores with both improving by more than two levels. While other improvements were seen in some domains of the CPCHILD and CCHQ, particularly comfort and communication, these showed wide confidence intervals. General improvements were seen in pain behaviour (PPP) at 12 weeks, while there were no changes seen for severity of dystonia (BADS).

**Table 3:** Secondary outcomes

Outcome measure	BaselineMean (SD)	6 weeksMean (SD)	12 weeksMean (SD)	Difference from baseline at 12 weeksMean 95 % (CI)
**COPM** performance	3.6 (0.9) *N* = 12	Not collected	7.0 (1.4) *N* = 11	3.5 (2.3, 4.6)
**COPM** satisfaction	2.2 (0.9) *N* = 12	Not collected	7.2 (2.6) *N* = 11	4.8 (3.1, 6.6)
**BADS**	23.8 (4.3) *N* = 12	20.9 (6.4) *N* = 10	21.9 (3.8) *N* = 10	-1.4 (-3.6, 0.8)
**FPS-R**	2.8 (1.4) *N* = 12	1.5 (1.3) *N* = 12	1.0 (1.3) *N* = 10	-1.8 (-3.1, -0.5)
**CPCHILD**				
Personal care	36.7 (20.3) *N* = 13	52.9 (22.3) *N* = 7	49.4 (18.2) *N* = 11	10.3 (2.0, 18.6)
Positioning/transferring/mobility	38.8 (19.7) *N* = 13	53.0 (16.8) *N* = 7	51.4 (17.9) *N* = 11	8.3 (-4.9, 21.6)
Comfort & emotions	62.4 (23.4) *N* = 13	78.9 (14.8) *N* = 7	81.2 (12.4) *N* = 11	11.1 (1.5, 20.8)
Communication/social interaction	54.6 (17.0) *N* = 13	72.1 (16.7) *N* = 7	70.0 (14.3) N-11	13.0 (2.6, 23.4)
Health	64.6 (17.7) *N* = 13	60.0 (13.9) *N* = 7	73.9 (11.7) *N* = 11	6.7 (-2.7, 16.1)
Overall QoL	67.7 (27.7) *N* = 13	80.0 (17.9) *N* = 7	70.9 (16.4) *N* = 11	0.0 (-6.0, 6.0)
Total	54.1 (16.6) *N* = 13	65.5 (14.0) *N* = 7	66.1 (11.7) *N* = 11	8.2 (0.9, 15.6)
**CCHQ**				
Personal care	4.1 (1.2) *N* = 13	2.6 (1.5) *N* = 6	3.2 (1.3) *N* = 11	-0.8 (-1.7, 0.10)
Positioning/transferring	3.9 (1.6) *N* = 13	2.3 (1.3) *N* = 6	2.5 (1.1) *N* = 11	-1.1 (-2.2, -0.10)
Comfort	3.6 (1.2) *N* = 13	2.7 (1.4) *N* = 6	2.5 (1.5) *N* = 11	-0.9 (-1.6, -0.20)
Interaction/communication	3.3 (1.1) *N* = 13	2.5 (0.9) *N* = 6	2.8 (1.1) *N* = 11	-0.5 (-0.9, -0.10)

*SD *Standard Deviation, *CI *Confidence interval, *COPM *Canadian Occupational Performance Measure, *BADS *Barry Albright Dystonia Scale, *FPS-R *Face Pain Scale- revised, *CPCHILD *Caregiver Priorities & Child Health Index of Life with Disabilities, *CCHQ *Care and Comfort Hypertonicity Questionnaire

## Discussion

This pilot study of gabapentin for pain management in children with dystonic CP showed that the feasibility of running a future randomised controlled trial is low largely because of incomplete data collection, reduced adherence to medication dosing and slow recruitment. Preliminary findings suggest gabapentin may improve pain behaviour, care and comfort and attainment of pain related goals; however, further research is required to confirm these findings.

Reduced adherence to medication dosing and incomplete data collection were common in this study. The dosing titration schedule was intentionally slow because children with dystonic cerebral palsy have higher comorbidities and are at an increased risk of having side effects with rapid up-titration. Clinical experience suggests they appear to be particularly sensitive to the sedating effects of gabapentin, which can then interfere with their functional activities and impact on compliance with the medication. Despite the slow titration, clinicians often adjusted the dose down once children experienced side effects and consequently few children reached the maximum dosage recommended. This dose adjustment is reflective of what happens in routine clinical care and highlights the need for further research to address dosage regimens related to potential side effects.

Incomplete data collection was primarily related to missing data in the medication logs or items on questionnaires. There is some level of uncertainty as to whether children missed doses of the medication or the data was simply not entered. Several questionnaires requiring parent completion were included in this pilot study to determine the most appropriate to use for future trials. Parents may not have been fully informed of questionnaire completion requirements, or the burden of the questionnaires may have been too great. Incomplete data was most noticeable at the 6-week assessment time point, highlighting the difficulty of collecting data at three close time intervals. In addition, several of the weekly PPP questionnaires were incomplete. Reducing the burden for participating families who are already time poor due to caring for a child with a disability is crucial. In future, online methods for recording adherence to the medication and completing questionnaires as well as reducing the number of questionnaires should be considered.

Recruitment was impacted by several factors highlighting the challenges in conducting trials in this population. The main barrier to recruitment was the high number of eligible children who were already prescribed gabapentin. In addition, a smaller number of children or parents/carers did not identify pain significant enough to meet eligibility criteria. Furthermore, children with predominant severe dystonia represent a small subset of the total CP population [19]; therefore creating a small recruitment pool. Neither willingness to participate nor side effects affected recruitment and retention. Only one child withdrew due to side effects related to dose, suggesting once recruited the majority of children completed the study.

Gabapentin is reported to have fewer side effects than other medications used for children with CP [20]. While it appeared to be generally well tolerated in this study with only one child withdrawing due to side effects of the medication, many children experienced side effects, with a number experiencing more than one. This was also the reason why many children did not reach the highest recommended dose. Consequently, although side effects or adverse events may not impact the ability to do a future trial with respect to numbers, it might impact on outcomes if effective doses cannot be reached.

Pain should be evaluated across a range of physical, social, and psychological constructs to accurately reflect pain in children with dystonic CP. The challenge is to choose a measurement battery that is feasible to complete and produces meaningful outcomes. A combination of self-report and parent proxy reported tools were included in this study to achieve the secondary aim of examining preliminary information on the effectiveness of gabapentin in reducing pain and dystonia, and improving comfort, wellbeing and attainment of occupational performance goals impacted by pain. These results clearly need to be interpreted with caution because the small sample size was not powered for effectiveness and there is large variation between children included. Despite this, the study provides some valuable preliminary information of a medication that targets pain, rather than targeting the dystonia with medications that have more severe unwanted side effects [9]. In addition, the results can be used to inform power calculations and will assist with the selection of responsive outcomes measures to assess efficacy of this intervention in any future trials.

Clinically important improvements were found in pain behaviour as reported by parents, performance and satisfaction of functional activities impacted by pain, and comfort. Small but clinically insignificant reductions in dystonia and self-reported pain were also found. Similar results were found in an observational study of gabapentin specifically targeting dystonia rather than pain in 69 children with improvements in pain and comfort, however that study reported a significant decrease in the severity of dystonia [10]. This decrease in dystonia might reflect the study’s higher dosages of gabapentin used, larger sample and significant proportion of children with a primary dystonia unrelated to CP.

Whilst this small feasibility study provides limited results on effectiveness, the sample size was adequate for the primary aim. A randomised controlled trial of gabapentin does not appear to be feasible in this population as the slow recruitment rate would impact significantly on its success. Careful selection of number, type and administration method of outcome measures and adjusted dosing schedules could improve data collection and medication adherence, however, future studies should consider alternative trial designs to answer questions around effectiveness of gabapentin for managing pain in children with dystonic CP. In addition, future research could focus on innovative methods for measuring pain in children who are unable to self-report due to communication and/or cognitive limitations.

In conclusion, chronic pain is common and impacts significantly on children with dystonic CP. Gabapentin may have the potential to reduce pain and thus improve the participation and quality of life of these children, however stronger evidence of its effectiveness is required utilising alternative trial designs due to the low feasibility of successfully conducting a randomised controlled trial.

## Data Availability

The datasets used and/or analysed during the current study are available from the corresponding author on reasonable request.
